# Designing an Indoor Air Quality Monitoring App for Asthma Management in Children: User-Centered Design Approach

**DOI:** 10.2196/27447

**Published:** 2021-09-22

**Authors:** Sunyoung Kim, Yunoh Park, Matthew K Ackerman

**Affiliations:** 1 School of Communication and Information Rutgers University New Brunswick, NJ United States; 2 Department of Computer Science Rutgers University New Brunswick, NJ United States

**Keywords:** asthma, children, indoor air quality, mobile app, smartphone, user-centered design

## Abstract

**Background:**

Indoor air pollution is a well-known risk factor that triggers and exacerbates asthma, the most common pediatric chronic disease. Using a mobile app to monitor indoor air quality could be promising in engaging children in keeping their indoor air quality clean and healthy as secondary environmental prevention for asthma management. However, no app is available to allow children to monitor, assess, and improve their indoor air quality.

**Objective:**

This study aims to design a mobile app that encourages children to monitor indoor air quality and track their asthma conditions through a user-centered, iterative design approach.

**Methods:**

We reviewed existing apps for indoor air quality monitoring or asthma management for children and conducted two sets of semistructured interviews with 12 children with asthma. We then iteratively created prototypes and evaluated and revised them.

**Results:**

Participants raised a series of outstanding questions on the prototype features and content that described their needs and perspectives, which informed the final designs. Following the identified requirements and recommendations, we developed two versions of the app: AirBuddy for presenting concrete information for indoor air quality and AirPet for gamifying the practice of monitoring indoor air quality.

**Conclusions:**

By following an iterative, user-centered design process, we developed two versions of an app to encourage children with asthma to monitor indoor air quality and track their asthma condition. The user-centered design approach revealed two crucial aspects that require deeper consideration when creating a child-friendly app, including balancing brevity and expressivity and considering the longitudinal effects of gamification. As a next step, we plan to conduct a longitudinal deployment study to evaluate the real-world effects of our apps.

## Introduction

### Background

Asthma is the most common pediatric chronic disease, characterized by recurrent attacks of breathlessness and wheezing. It is estimated that more than 340 million people have asthma globally [1], and 8.4% of children in the United States had asthma in 2018 [2]. Childhood asthma creates a substantial burden on the affected children and their families by requiring regular medical encounters, restricting the child’s activities, and increasing the risk of school absences [3].

While asthma cannot currently be cured, it can be controlled through improved health care and avoiding or reducing asthma triggers from environmental factors. Among various environmental factors that contribute to excessive asthma morbidity, exposures to air pollutants are a crucial contributor to worsening the symptoms [4-6]. The relationship between fine particulate matter and asthma morbidity is especially well established [7]. Because children spend most of their time indoors, indoor environments dominate exposures to many air pollutants [8]. Thus, it is important to monitor indoor air quality (IAQ) for asthma management [9]. However, childhood asthma management is challenging because it requires understanding the causes of triggers and avoiding them, with triggers being both multifactorial and unique to each individual [10]. Moreover, it is difficult for doctors and parents to monitor the health of children with asthma simultaneously with environmental triggers.

Smartphones are ubiquitous, sensors have become prominently used in mobile health (mHealth), and mobile apps offer new opportunities for access to care and monitoring and managing a chronic disease [11,12]. mHealth apps provide various features to facilitate asthma management, including medication reminders, symptom monitoring, prompt communication with a provider, and access to tailored education, information, and resources [13-15]. Because asthma is a chronic condition, it is crucial to educate affected children on how to live with it, and mobile apps can meet such needs. However, few apps offer ways to monitor air pollutants indoors, some of the most frequent triggers for asthma attacks, and even fewer apps are specifically designed for children. For mobile apps to successfully manage chronic diseases, designers must be committed to user-centered, evidence-based design to meet user needs. Beyond the requirements of good design and development, the central question of designing mobile apps for children with asthma is how to accommodate the perspectives and needs of children and engage children in digital interactions that foster positive outcomes for asthma management.

### Objectives

The objectives of this study were to investigate children’s perspectives and needs in the design of a child-friendly mobile app and develop, through a user-centered, iterative design approach, a mobile app that encourages children to monitor IAQ.

## Methods

### Overview

User-centered design is an iterative process in which designers focus on users and their needs in each phase of the design process by putting users at the center of product development [16]. General steps in user-centered design include evaluating and applying relevant theory, understanding user needs and the environment in which the app will be used, and iteratively producing and evaluating prototypes for the final product design [16]. This study followed the user-centered process to iteratively design and refine a child-friendly IAQ monitoring app for asthma management.

### Participant Recruitment

Children aged 8 to 12 years with moderate to severe persistent asthma, as determined by the National Institutes of Health guidelines for the diagnosis and management of asthma [17], were eligible to participate in the study. We chose the 8- to 12-year age range because children around the age of 8 years start to understand basic terms and sentences and shift from learning to read to reading to learn, and thus they can use digital devices for autonomous tasks [18]. Since we planned to design a mobile app that provides IAQ information in simple written sentences to help children understand IAQ, we targeted children with asthma who can read simple sentences. Thus, children were not eligible if they could not read or speak English or if their involvement was deemed inappropriate by the pediatrician because of their mental and physical conditions.

After obtaining the institutional review board’s approval, we recruited participants from a children’s hospital and school-based health centers in an urban area. For recruitment, we first contacted pediatricians and explained the purpose of the study and recruitment criteria to them. The pediatricians recommended potential participants among their patients (upon guardian approval) who regularly visit the hospital. We then contacted guardians of children with asthma by telephone. A total of 12 guardians whose children met the inclusion criteria were contacted for recruitment. All of their children were recruited to participate in the study (6 females and 6 males, mean age 9.8 [SD 1.6] years; Table 1). After we obtained temporary consent to participate in the study by telephone, guardians and their children provided consent electronically during the interview.

**Table 1:** Participant demographics.

ID	Age (years)	Gender	Ethnicity	Asthma severity	Guardian’s relationship to participant	Guardian’s highest educational attainment
P1	12	M	Hispanic	Intermittent	Mother	2-year college
P2	9	F	Hispanic	Intermittent	Grandmother	Less than high school
P3	10	M	White	Intermittent	Mother	2-year college
P4	12	M	White	Intermittent	Mother	2-year college
P5	11	M	White	Moderate	Mother	University
P6	8	M	White	Intermittent	Mother	Graduate
P7	8	F	White	Intermittent	Mother	University
P8	8	F	White	Intermittent	Mother	University
P9	10	F	White	Intermittent	Grandmother	High school
P10	8	M	Black	Moderate	Mother	University
P11	11	F	White	Mild	Mother	University
P12	11	F	Black	Mild	Mother	2-year college

### Data Collection

#### Reviewing Existing Apps

**Figure 1:**
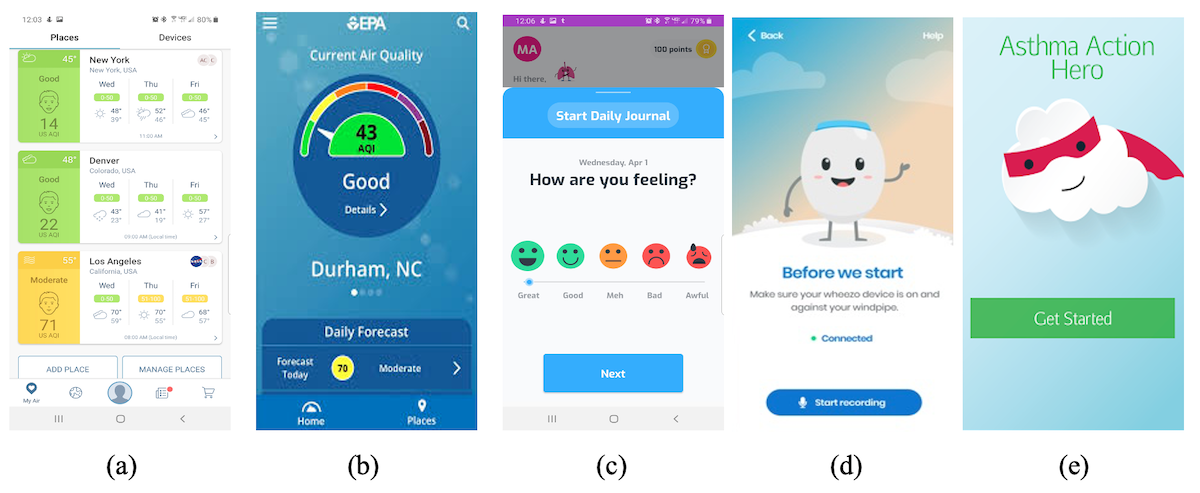
Screenshots of apps found during app review: (a) AirVisual, (b) AirNow, (c) AsthMe, (d) Wheezo, and (e) AsthmaActionHero.

**Figure 2:**
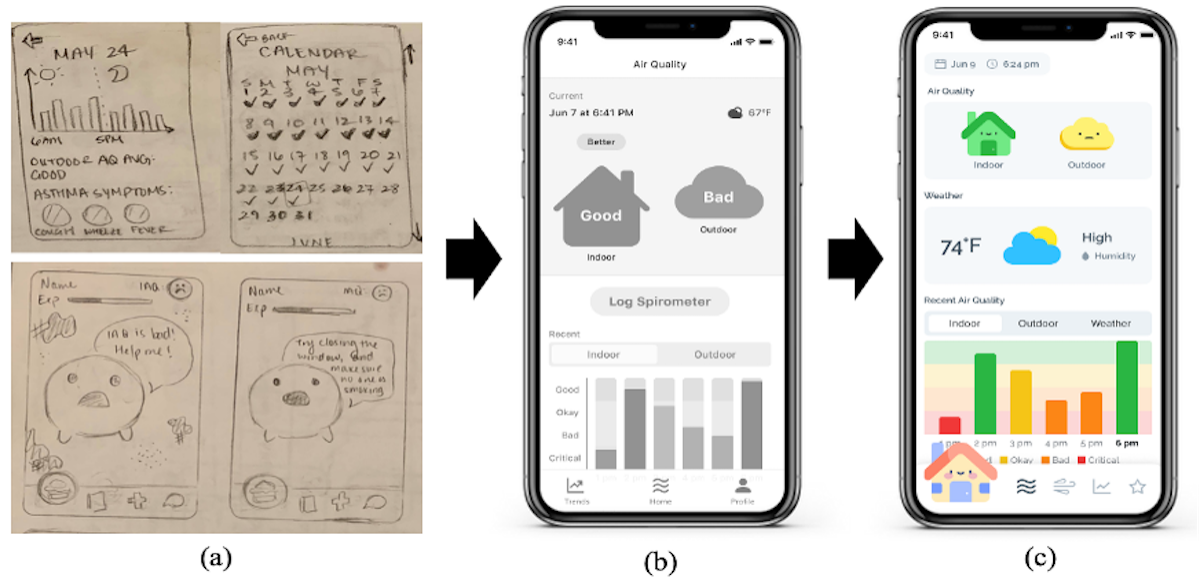
Prototyping process of AirBuddy that used emojis and a bar graph from (a) sketches to a (b) wireframe to a (c) high-fidelity prototype.

**Figure 3:**
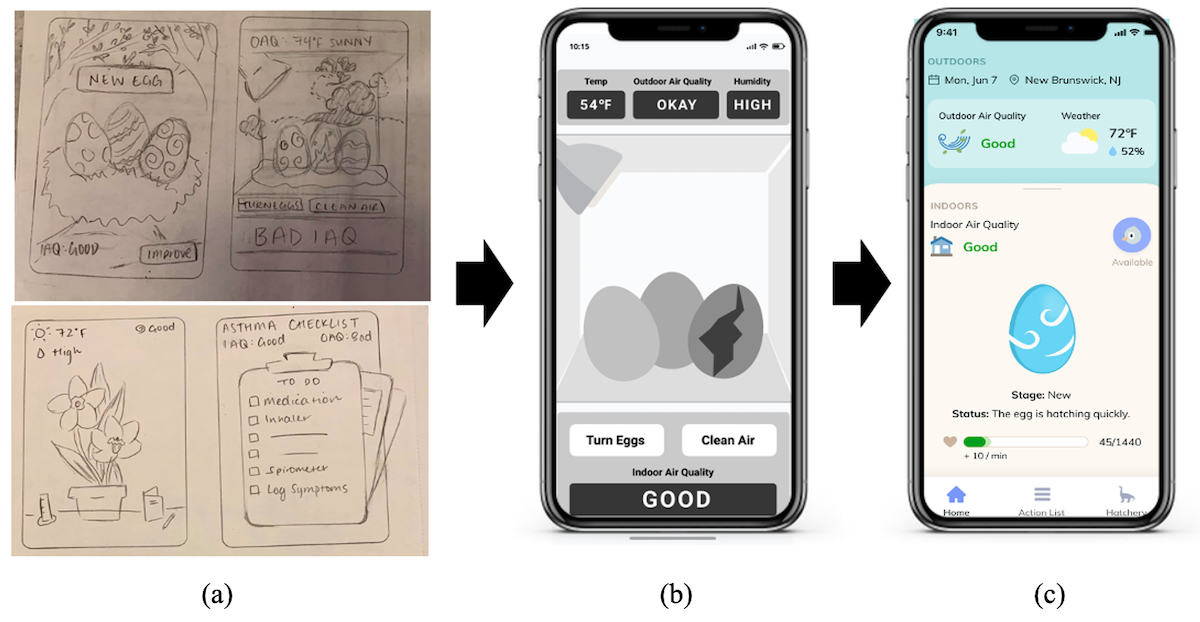
Prototyping process of AirPet, a gamified app using egg hatching to display indoor air quality from (a) sketches to a (b) wireframe to a (c) high-fidelity prototype.

We conducted a review of mobile apps available in the market that offer functionalities on IAQ monitoring or asthma management for children to establish baseline concepts and functionalities for our app. While we conducted an extensive review of existing apps available on Apple’s App Store and Google Play, we did not find any app that offered functionalities for IAQ monitoring associated with asthma management or IAQ monitoring for children. Thus, we selected instead two IAQ monitoring apps, AirNow and AirVisual, and three asthma management apps designed for children—Wheezo, a digital stethoscope that records breathing sounds to detect signs of asthma; AsthMe, an information repository for asthma management; and AsthmaActionHero, a mobile diary to record asthma conditions and take actions for asthma management—based on the number of downloads, reviews, and average user ratings (Figure 1). Based on a review of existing apps, we created two sets of low-fidelity sketch prototypes for our app (Figures 2 and 3).

#### Understanding User Needs

An important step in user-centered design is to establish a basic understanding of the practices and needs of end users to guide the ideation and design of an app. To obtain this, we conducted semistructured interviews with each child. For the interviews, we created a set of open-ended interview questions with 3 key themes: (1) exploring how children with asthma manage and live with their conditions, (2) investigating the experiences of using mobile apps in general and for asthma management in particular, and (3) learning about the current understanding of IAQ and its relationship to asthma. In addition, we collected participant basic demographic information including age, gender, and asthma severity from their guardians.

All interviews were done virtually using videoconferencing software of the participant’s choice (eg, Skype, Zoom). While all questions were to be asked to children, we also encouraged participant’s guardians to join the interview and share their thoughts about the question or their child’s answer when they wanted. Last, we displayed our low-fidelity sketch prototypes on a shared screen, explained the key concepts of each sketch, and asked the participants for feedback. Each interview lasted about 30 minutes.

#### Iterative Design and Evaluation

Based on our prototype sketches, collected requirements, and feedback in the previous steps, we iteratively created a set of wireframes that were then developed into high-fidelity prototypes (Figures 2 and 3). We conducted one-on-one interviews with the same group of participants to receive feedback on the prototypes. In the interview, we first introduced our app to participants as “a mobile app that allows you to monitor air qualities in your home and outside, record your asthma conditions, and find information about actions to take for asthma management.” We displayed our prototypes on a shared screen and asked participants to think aloud and express their thoughts and feelings while freely exploring the prototypes as much as they wanted.

During the interview, a participant verbally commanded their intended interactions with a prototype cast on a shared screen. Then, an interviewer executed the user’s interaction commands as a form of the Wizard of Oz study. When participants had no more ideas to share, we asked 3 questions about each screen based on the cognitive walkthrough method, including (1) visibility, did the user notice the correct action to take; (2) affordance, did the user associate the correct action with the outcome they expect to achieve; and (3) feedback, if the correct action was performed, did the user see that progress was being made toward their intended outcome [19]. Each interview lasted up to 60 minutes. After completing this interview, the guardians of participants were sent an electronic gift card for their child’s participation.

### Data Analysis

We analyzed the interview data using thematic analysis to reveal patterns across data sets and draw out significant themes [20]. The emerging themes were continuously discussed by the authors until data were saturated and no new information was anticipated. First, we conducted open coding to identify concepts that were significant in the data. The following example shows that one participant did not understand how the egg hatching concept relates to IAQ, and this response was coded as “unclear concept.”

{Unclear concept} What does the air quality inside have to do with hatching an egg? [Participant P2]{/Unclear concept}

We then categorized the related concepts created by open coding using axial coding. Phenomena refer to repeated patterns of events, happenings, actions, and interactions representing people’s responses to problems and situations. For instance, “confusion” is a phenomenon that refers to a participant’s incorrect or lack of understanding about the meanings of the visual representation in the app interface. During axial coding, the open code “unclear concept” in the example above was categorized as “confusion” since the participant did not understand the meaning of egg hatching. Last, we performed the selective coding process to integrate the categories derived from axial coding.

## Results

### Design Conceptualization

From the review of the existing apps, 4 trends emerged that were prevalent or common across apps. First, all apps used vivid colors and simple graph components for information visualization (Figure 1a). For instance, the apps for air quality monitoring used colored graphical components such as speedometers, emojis, and bar graphs with color scales to indicate the level of air quality (Figure 1b). In contrast, the apps for asthma management allowed users to pick different emojis and other colored graphical components to indicate the user’s condition (Figure 1c). Second, all apps used graphic characters and personified graphical components to make information more engaging and fun (Figure 1d and 1e). Third, all apps provided the features to track the history or trend of the monitored information. For instance, graphs were used by apps for air quality monitoring to keep track of the changes in air quality indoors and outdoors over time, and the apps for asthma management offered the features to record and keep track of asthma symptoms and conditions. Last, all apps offered informational content to encourage users to make beneficial, real-life changes. For instance, the apps for air quality monitoring provided information about how users should respond to poor air quality indoors. And the apps for asthma management provided information about how to prevent or react to asthma symptoms.

With these themes, we came up with 2 design concepts and created 2 corresponding sets of sketch prototypes to meet the objectives of our app in different ways. The first concept was a conventional form of concrete information presentation using graphical components such as graphs, calendars, and emojis to present the level of air qualities indoors and outdoors and allow logging the user’s asthma condition (Figure 2a). The second concept was gamification using the theme of raising a pet, in which the levels of IAQ and user engagement with an app turn into treats to feed a virtual pet. For a virtual pet, we chose 2 themes, egg hatching and planting (Figure 3a).

### User Needs and Feedback on Sketch Prototypes

All participants had a decent understanding of asthma, what triggers asthma attacks, and what to do when asthma flares up. Regarding asthma triggers, all participants mentioned humidity and heat (weather) as primary causes of symptom worsening, whereas there was no understanding of the relationship between air pollution and asthma. At best, air pollution was considered analogous to humidity to some participants. Most participants did not have much interest in or knowledge about air quality or its effects on asthma. These show the importance of providing IAQ information along with weather and humidity to help users understand the relationships among temperature, humidity, IAQ, and asthma.

Asthma affects your breathing and makes it harder to breathe. Like when it gets really hot, or in my hot showers when the room gets steamy, I have trouble breathing.

Participant P2

A few days ago, when I was walking with my mom, my dad, and my brother, the air was getting humid and dense, and it was getting hard for me to breathe. I think humidity makes it hard to breathe, harder to breathe sometimes.

Participant P8

Like probably more polluted air, like more humid air affects me more than just fine air.

Participant P3

The primary resources of information for asthma management were parents and the internet. None of the participants was using an app for asthma management or expressed any interest in using one, which shows the importance of actively motivating user engagement with the app.

When I have any question, my mom and dad are the first choice. If that doesn’t work, probably the internet because I’m not always at the doctor’s office.

Participant P3

I usually ask Alexa for how the weather is, like what’s the weather today.

Participant P4

When we showed our sketch prototypes to participants during the first interview, the overall response was positive on both prototypes, although they did not provide detailed feedback. It might be because the low-fidelity prototypes missed many details making it difficult for children to offer exhaustive responses. All participants confirmed that both themes were easy to understand. For sketch prototypes with traditional graphical components, participants expressed equal preferences on a bar graph, a calendar, and emojis. For sketch prototypes with gamified concepts, participants expressed a preference for egg hatching over planting.

### Iterative Design for High-Fidelity Prototyping

**Figure 4:**
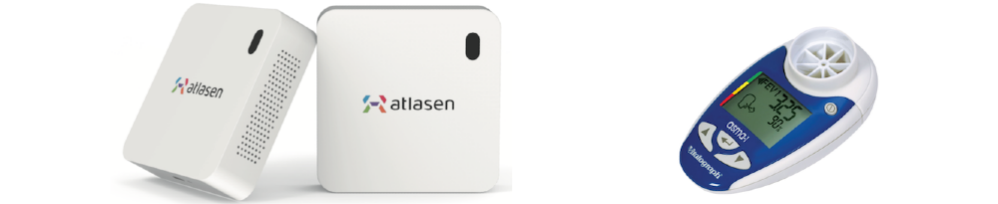
Images of an indoor air quality sensor that measures PM 2.5, CO_2_, and NO_2_ (left) and a spirometer that measures FEV1, FEV6, and % of personal best FEV1 (right).

Based on user feedback on the sketch prototypes and their expressed needs, we iteratively created 2 sets of wireframes, which we developed into high-fidelity prototypes. Key features of the app include visualizing air qualities indoors and outdoors in real time and keeping track of the user’s asthma condition. In the system development, IAQ data will be transmitted from a separate IAQ sensor (Figure 4, left). The outdoor air quality data will be retrieved from an AirNow application programming interface that provides current air quality data by zip code. A commercialized spirometer will be provided to participants to measure lung condition and enter results into the apps (Figure 4, right). An IAQ sensor and spirometer will be packaged with the app.

**Figure 5:**
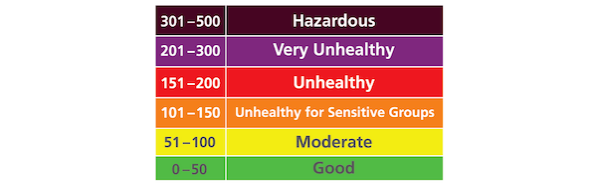
Air Quality Index color codes for Particulate Matters.

The first wireframe, which we named AirBuddy, was created using a combination of a bar graph and emojis to visualize air quality indoors and outdoors (Figure 2b). Through several design iterations and discussions within the research team, we developed the wireframe into a high-fidelity prototype that displays color-coded house and cloud icons to show the current air quality levels indoors and outdoors, respectively. Also included are weather forecast, humidity, and a color-coded bar graph presenting the weekly trend of these data (Figure 2c). We juxtaposed a house icon with a cloud icon so that users can compare air quality indoors and outdoors. For the color codes, we used the US Environmental Protection Agency’s Air Quality Index (AQI) color codes to visually present the level of air quality (Figure 5). For a feature for engagement, this prototype provides a personified house character, Airic, a chatbot placed at the bottom left corner of the navigation bar. It answers questions regarding air quality and asthma in simple language suitable for children and reminds them to enter spirometer readings to the app.

The second wireframe, named AirPet, was created using a gamification theme of egg hatching (Figure 3b). We developed the wireframe into a high-fidelity prototype through several design iterations and discussions within the research team that shows a virtual egg for which hatching speed is determined by the level of air quality indoors and daily spirometer data entry (Figure 3c). This prototype uses the status of IAQ and whether or not daily spirometer data is recorded as factors to affect the speed of egg hatching, and the hatching progress is displayed on a progress bar underneath the egg. Maintaining good IAQ and recording daily spirometer data will hatch approximately one egg a week. In addition, air quality outdoors and weather information are displayed on top of the screen.

Since the concepts of these 2 prototypes are entirely different—AirBuddy for concrete information presentation and AirPet for abstract gamification—representing different aspects of strengths and weaknesses in the interface design, we decided to keep both ideas for the final system design.

### User Feedback on High-Fidelity Prototypes

The first few comments across all participants about our prototypes indicated preferences toward colorful interfaces, which confirms prior work showing that color is a distinguishing characteristic for children’s engagement with a digital interface [21]. Participants were all attracted to both apps for their colorful interfaces and positively mentioned most graphical components provided in the app.

Both have popping colors. I think it helps to get its attention.

Participant P5

I like it (AirPet). It is beautiful.

Participant P10

The house [in AirBuddy] can talk! If I wanted to say, “Hi house,” what would it say back? Hi? Can I try it?

Participant P11

While vivid colors were an effective component to draw children’s attention to the app, we found it was not adequate to use color to convey information. The meanings of colors in the AQI color codes were green for good air quality, orange for moderate air quality, red for unhealthy air quality, and purple for extremely unhealthy air quality. When we asked participants how they interpreted the meanings of different colors concerning the level of air quality in the app, however, many participants interpreted purple as better air quality than when it was red. This suggests that care must be taken when using colors to convey information in a child-friendly user interface or users might misinterpret the information.

I think the red will stand for the highest bad air and then the purple will be a lower level and the orange will be lower and yellow will be lower and green will be the best time to go outside.

Participant P7

The colors? Green means good. Yellow means okay. Orange means getting kind of good. Pink means kind of bad. And then, kind of very bad for purple. And then, red is very bad.

Participant P9

The colors of house and cloud icons in the AirBuddy were designed to change based on air quality levels indoors and outdoors to present them visually. However, many participants did not know how to interpret these colored icons until we explained them in detail.

The cloud makes me think of the air and stuff around. The house is just… I don’t know. It doesn’t really make me think of air quality. It makes me think just the house.

Participant P1

I think the cloud is for the air quality inside, but I am not sure what the house is. Maybe adding something to explain what this means will be helpful.

Participant P9

In addition, we found that a color-coded bar graph in AirBuddy to present the recent trend of air quality indoors and outdoors influenced participants’ perspectives on the app both positively and negatively. The positive aspect was that most participants easily understood the concept of a bar graph because they were familiar with it from other contexts, such as in class or for asthma measurement. The negative aspect was that those different contexts where a bar graph was used made participants perceive the app as less fun or engaging and instead more educational and informational.

Because of this (a bar graph), it (AirBuddy) looks like a grading type of app or something that I use in school that gives you a report on how you do.

Participant P7

It (AirBuddy) looks familiar. We use something like that in science at school and also the thing that I see when I have to breathe into the thing, and it shows how long I can breathe.

Participant P12

In the design of AirPet, we applied the concept of egg hatching to present IAQ information simply yet effectively, which turned out to be a positive factor for participants’ initial impression of the app. Participants expressed their preference for AirPet over AirBuddy because AirPet’s gamification factors made interaction with the app more engaging and fun. However, the concept of egg hatching related to IAQ and the progress bar was confusing and unclear to some participants.

I like this app (AirPet) better because it gives you a better experience on how to learn about air quality and how asthma works and how to get rid of it.

Participant P6

I think that the air inside is what helps you hatch the egg. The better the air, the better the air hatches.

Participant P7

I think the egg would represent the air and how you make it better. By making the egg better, you are making your asthma better.

Participant P10

What does the air quality inside have to do with hatching an egg? And, what are all these numbers underneath the egg?

Participant P2

Last, participants’ parents expressed positivity toward the utility of the apps for easy access to information and fostering a child’s skill for asthma self-management.

I think any way that you can take some ownership over your own health issues is definitely a beneficial thing, especially since he [her son] has massive amounts of food allergies, which also is part of what triggers his asthma. So, I think, any way that he can have tools to help him where I am not micromanaging his health would definitely be good.

Participant 6’s parent

Sometimes you are not quite sure what could be harming your child or what could help them. And if you go online, there is tons of information, but I think having it broken up by category with very concrete examples is helpful.

Participant 7’s parent

### Final Design

**Figure 6:**
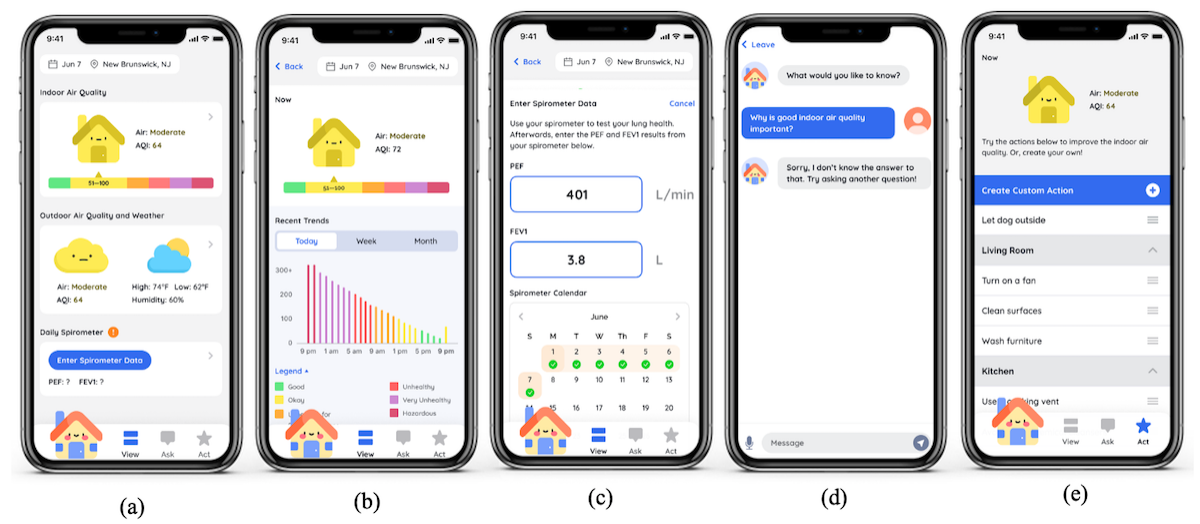
Final design of AirBuddy: (a) home screen, (b) detailed indoor air quality information, (c) spirometry entry page, (d) chatbot Airic, and (e) list of action items to improve indoor air quality.

The biggest concern with the AirBuddy prototype was that the interface was too simple to convey IAQ information effectively because it relied solely on the shape and color of an icon. To address this issue, we separated a house icon from a cloud icon and located a horizontal AQI color strip underneath the house icon to indicate the current IAQ. We also added text to display a label and numerical level of IAQ next to the house icon (Figure 6a). When a user clicks anywhere in the IAQ information pane on a home screen, the app moves to an IAQ detail page where a bar graph of the recent IAQ trends is located. We moved the bar graph to a subpage so that the app can still benefit from the target users’ familiarity with a bar graph to convey the IAQ trend information but reduce its influence on the overall and initial perception of the app (Figure 6b).

Next, we grouped a cloud icon with other weather conditions to conceptualize outdoor air quality as part of outdoor/weather information. Underneath the weather pane is a button to log daily spirometer data. Clicking this button brings up a subpage where a user can enter spirometer readings and review the daily log of previous data entry (Figure 6c). Clicking the house icon at the bottom left corner of a navigation bar will bring up a chatting page where a user can interact with Airic, a chatbot, both verbally and via typing, to ask any question relating to IAQ and asthma management (Figure 6d). Last, we provided a list of action items that the user can perform to improve IAQ (Figure 6e).

**Figure 7:**
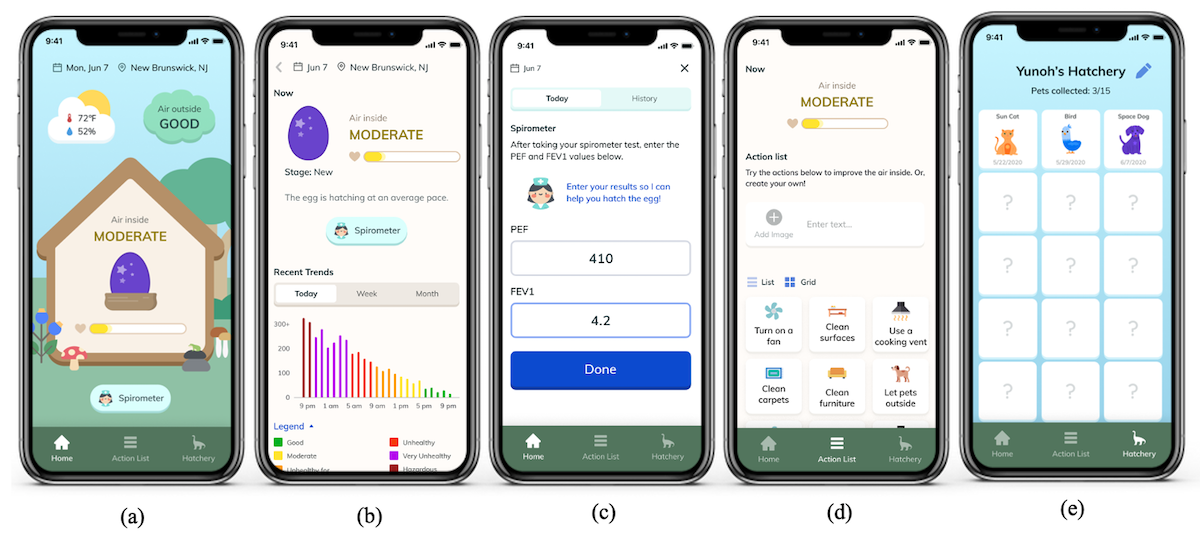
Final design of AirPet: (a) home screen, (b) detailed indoor air quality information, (c) spirometry entry, (d) list of action items to improve indoor air quality, and (e) hatchery with hatched pets.

The biggest concern with the AirPet prototype was that the link between the concepts of egg hatching and IAQ could be confusing to some users. We redesigned the home screen to illustrate an egg that a house broods to hatch graphically to address this issue. We also simplified the progress bar and added text to display a labeled level of IAQ inside the home. In addition, we added two clouds outside the house to represent the level of air quality outdoors and weather information (Figure 7a). When a user clicks anywhere in the house on a home screen, the app moves to an IAQ detail page where we located a bar graph adopted from AirBuddy to convey the IAQ trend information (Figure 7b).

Underneath the house on the home screen is a button to log daily spirometer data. Clicking this button or the same button on an IAQ detail page will bring up a subpage where a user can enter spirometer readings and review the daily log of previous data entry (Figure 7c). Last, we provided a list of action items a user can perform to improve IAQ and a list of virtual pets that were successfully hatched (Figures 7d and 7e).

## Discussion

### Principal Findings

Indoor air pollution is a known risk factor contributing significantly to adverse respiratory health in children [22]. Thus, keeping IAQ clean and healthy is the basis of secondary environmental prevention for asthma management [23]. However, our study showed that the affected children had little or no knowledge about indoor air pollution or how it affects their asthma symptoms [24]. While parents are and need to be the primary source of information for their children’s asthma management, the affected children must have a method for self-management of asthma. This suggests a need for an easy yet effective mechanism that helps ensure children’s engagement with IAQ as part of asthma management.

In this study, we conducted a series of interviews to involve the end users, children with asthma, throughout the process of designing an app that promotes their engagement in monitoring and improving IAQ and tracking daily asthma conditions through a user-centered design approach. We iteratively revised and improved the prototypes through this process to ensure that children can use our app easily, effectively, and reliably. This process has revealed 2 crucial aspects that require deeper consideration when creating a child-friendly app, including balancing brevity and expressivity and considering the longitudinal effects of gamification.

### Balancing Simplicity and Expressivity

A central consideration in the design of our app was how to present IAQ information simply yet effectively so that children can easily understand, engage in, and play with it toward improving IAQ. The prevalent mode of presenting the level of air quality is to use AQI values with associated color codes (green: 0-50 for good; yellow: 51-100 for moderate; orange: 101-150 for unhealthy for sensitive groups; red: 151-200 for unhealthy; violet: 201-300 for very unhealthy; and purple: 301-500 for hazardous). We adopted only the AQI color codes to present the air quality status in the early version of the AirBuddy prototype because providing numeric values might be too complicated for children to understand. The research team concluded that the interface would be simple enough for children to use.

Our assumption was wrong in 2 aspects. First, the findings showed that this prototype was too simple and brief for children of our target age group who can handle and even seek out complex information to interpret and contextualize the meaning. For instance, many participants did not understand how to interpret colored icons without detailed explanations. While focusing on simplicity, we failed to deliver enough information to visually present the air quality levels effectively. Second, the findings showed that using color is powerful for children’s engagement but might not be effective in conveying meaningful information. Unlike adults who are conscious of the symbolic meanings of colors, children have yet to acquire the contextual information associated with different colors. Thus, care should be taken when using colors to convey information to children.

These findings demonstrate that special attention should be paid to assure a good balance between simplicity for ease of use and expressivity for effective delivery of information when designing a child-friendly interface. A central tenet for user-centered design practices is that one size does not fit all, but the design should be driven by the knowledge and perspectives of the target users [16]. The iterative, user-centered design process with our participants made clear that our prototype was simple enough for children to play with but not expressive enough for representing information, which could not have been identified without the user-centered design process.

### Longitudinal Effects of Gamification

Gamification is defined as the use of game design elements in nongame contexts [25]. This principle has been used in a variety of contexts for children, including but not limited to education [26], behavior change [27], and health care [28], to make tedious activities more engaging to children and thus increasing their motivation to use them [29]. Because autonomous and regular engagement with the app is crucial to meet the goal of our app, we adopted gamification elements in AirPet in which a user must regularly access the app to monitor and improve IAQ and log asthma conditions to hatch a virtual pet. The overall responses from participants were positive, and most of them preferred this prototype over the other one, thanks to the gamification elements. Thus, we are hopeful that the gamification elements would positively influence children’s sustained engagement with our app.

However, the actual and, especially, longitudinal effects of gamification on children’s engagement are controversial. Numerous apps have been using gamification for children’s engagement. And some studies even demonstrated that gamification is statistically effective in improving children’s engagement [30]. But other studies showed that the effect of gamification might fade away [31] or even negatively influence motivation, satisfaction, and empowerment as the use duration increases [32]. This suggests that gamification is not a panacea for all. Designers need to be cautious of potential negative effects when applying certain gamification mechanics for sustained engagement. In the next step, we plan to conduct a field deployment study using the apps we designed to investigate how gamification, in contrast to a conventional form of concrete information presentation, influences children’s engagement in IAQ and asthma management over time.

### Limitations

Our original plan was to conduct participatory design workshops for idea exploration and run in-person interviews to evaluate an interactive high-fidelity prototype. However, due to the CDC recommendations for social distancing during COVID-19, we canceled the workshops and instead conducted all interviews one-on-one virtually.

Our findings must be evaluated within the context of several limitations. First, our sample size was small, and thus our participant pool may not represent a general population. Second, we used convenience sampling by recruiting participants from a children’s hospital and school-based health centers in an urban area, which also runs the risk of compromising generalizability. Selection bias or unmeasured factors (eg, the homogeneity of participant characteristics by living in the same geographic regions) could have influenced the responses during the interviews. Third, we conducted all interviews virtually due to the pandemic, which might have affected the participants’ experience with our prototypes differently from how they would experience them with direct interaction. We are hopeful that we can conduct in-person interviews in a system deployment study planned as the next step of this project.

### Conclusion

This work completes the foundational stages of concept generation, iterative design, and implementation in the user-centered design process. These stages are fundamental to the subsequent evaluation and deployment of the app to support children with asthma to monitor and improve IAQ. The next phase is to conduct usability testing of a working system with end users to evaluate its effectiveness for children’s use before public deployment. Our iterative design process demonstrated that it is critical to engage potential users as early as the concept generation phase and throughout the iterative design stages to assure that the final app meets user needs as intended. A similar user-centered design approach can be effectively applied in the development and design of mHealth apps to address self-management needs for other pediatric conditions. As a next step, we plan to conduct a longitudinal deployment study with children with asthma to evaluate the real-world effects of our apps. We will investigate how different approaches—a conventional form of concrete information presentation and abstract gamification—influence the affected children’s engagement with IAQ and how the increased awareness of IAQ influences IAQ and asthma conditions.

## Abbreviations

AQI: Air Quality Index
IAQ: indoor air quality
mHealth: mobile health

## References

[1] GBD 2016 Disease and Injury Incidence and Prevalence Collaborators (2017). Global, regional, and national incidence, prevalence, and years lived with disability for 328 diseases and injuries for 195 countries, 1990-2016: a systematic analysis for the Global Burden of Disease Study 2016. Lancet.

[2] (2018). Asthma Surveillance Data.

[3] Ramratnam SK, Bacharier LB, Guilbert TW (2017). Severe asthma in children. J Allergy Clin Immunol Pract.

[4] D'Amato G, Liccardi G, D'Amato M, Holgate S (2005). Environmental risk factors and allergic bronchial asthma. Clin Exp Allergy.

[5] Erbas B, Kelly A, Physick B, Code C, Edwards M (2005). Air pollution and childhood asthma emergency hospital admissions: estimating intra-city regional variations. Int J Environ Health Res.

[6] Guo H, Huang S, Chen M (2018). Air pollutants and asthma patient visits: indication of source influence. Sci Total Environ.

[7] Garcia E, Berhane KT, Islam T, McConnell R, Urman R, Chen Z, Gilliland FD (2019). Association of changes in air quality with incident asthma in children in California, 1993-2014. JAMA.

[8] Franklin PJ (2007). Indoor air quality and respiratory health of children. Paediatr Respir Rev.

[9] Akar-Ghibril N, Phipatanakul W (2020). The indoor environment and childhood asthma. Curr Allergy Asthma Rep.

[10] Martinez FD (2009). Managing childhood asthma: challenge of preventing exacerbations. Pediatrics.

[11] Anderson K, Burford O, Emmerton L (2016). App chronic disease checklist: protocol to evaluate mobile apps for chronic disease self-management. JMIR Res Protoc.

[12] Ranjan Y, Rashid Z, Stewart C, Conde P, Begale M, Verbeeck D, Boettcher S, Dobson R, Folarin A, RADAR-CNS Consortium (2019). RADAR-Base: open source mobile health platform for collecting, monitoring, and analyzing data using sensors, wearables, and mobile devices. JMIR Mhealth Uhealth.

[13] Camacho-Rivera M, Vo H, Huang X, Lau J, Lawal A, Kawaguchi A (2020). Evaluating asthma mobile apps to improve asthma self-management: user ratings and sentiment analysis of publicly available apps. JMIR Mhealth Uhealth.

[14] Ramsey RR, Plevinsky JM, Kollin SR, Gibler RC, Guilbert TW, Hommel KA (2020). Systematic review of digital interventions for pediatric asthma management. J Allergy Clin Immunol Pract.

[15] Tinschert P, Jakob R, Barata F, Kramer J, Kowatsch T (2017). The potential of mobile apps for improving asthma self-management: a review of publicly available and well-adopted asthma apps. JMIR Mhealth Uhealth.

[16] Abras C, Maloney-Krichmar D, Preece J, Bainbridge W (2004). User-centered design. Encyclopedia of Human-Computer Interaction.

[17] National Asthma Education and Prevention Program (NAEPP) Coordinating Committee (CC) (2012). Expert Panel Report 3: Guidelines for the diagnosis and management of asthma 2007.

[18] Markopoulos P, Bekker M (2003). Interaction design and children. Interacting with Computers.

[19] Rieman J, Franzke M, Redmiles D (1995). Usability evaluation with the cognitive walkthrough. Conf Companion Human Factors Comput Syst.

[20] Braun V, Clarke V (2006). Using thematic analysis in psychology. Qual Res Psychol.

[21] Large JA, Beheshti J (2005). Interface design, web portals, and children. Library Trends.

[22] Franklin PJ (2007). Indoor air quality and respiratory health of children. Paediatr Respir Rev.

[23] Gautier C, Charpin D (2017). Environmental triggers and avoidance in the management of asthma. J Asthma Allergy.

[24] Kim S, Senick JA, Mainelis G (2019). Sensing the invisible: understanding the perception of indoor air quality among children in low-income families. Int J Child Comput Interact.

[25] Deterding S, Dan D, Khaled R, Nacke L (2011). From game design elements to gamefulness: defining gamification. Proc 15th Int Academic MindTrek Conf.

[26] Caponetto I, Earp J, Ott M (2014). Gamification and education: a literature review. Proc 8th Eur Conf Games-Based Learn.

[27] Cugelman B (2013). Gamification: what it is and why it matters to digital health behavior change developers. JMIR Serious Games.

[28] Miller AS, Cafazzo JA, Seto E (2016). A game plan: gamification design principles in mHealth applications for chronic disease management. Health Informatics J.

[29] Pramana G, Parmanto B, Lomas J, Lindhiem O, Kendall PC, Silk J (2018). Using mobile health gamification to facilitate cognitive behavioral therapy skills practice in child anxiety treatment: open clinical trial. JMIR Serious Games.

[30] Fadhli M, Brick B, Setyosari P, Ulfa S, Kuswandi D (2020). A meta-analysis of selected studies on the effectiveness of gamification method for children. Int J Instruction.

[31] Mavletova A (2015). A gamification effect in longitudinal web surveys among children and adolescents. Int J Market Res.

[32] Hanus MD, Fox J (2015). Assessing the effects of gamification in the classroom: a longitudinal study on intrinsic motivation, social comparison, satisfaction, effort, and academic performance. Comput Educ.

